# Trans-cutaneous electrical nerve stimulation to treat dry mouth (xerostomia) following radiotherapy for head and neck cancer. A systematic review

**DOI:** 10.1016/j.amsu.2021.01.094

**Published:** 2021-02-03

**Authors:** Fatemeh Salimi, Francisco Saavedra, Brain Andrews, James FitzGerald, Stuart C. Winter

**Affiliations:** a Surgical Science Department, University of Oxford, United Kingdom; b Electrical Engineering Department, Faculty of Engineering, University of Concepcion, Chile; c Nuffield Department of Surgical Science, University of Oxford, United Kingdom

**Keywords:** Xerostomia, Head and neck cancer, Dry mouth in patients with head and neck cancer, Post radiotherapy complications in patients with head and neck cancer, Transcutaneous Electrical Nerve stimulation of the salivary glands, TENS machine in production of sa

## Abstract

**Background:**

A dry mouth or xerostomia is one of the most common long-term complications following radiotherapy for head and neck cancer and has a negative impact on quality of life in cancer survivors. Transcutaneous electrical nerve stimulation (TENS) is a novel approach to improving saliva flow in these patients.

**Objective:**

To perform a systematic review of studies evaluating TENS in the treatment of radiotherapy induced xerostomia in head and neck cancer patients.

**Data collection and analysis:**

A comprehensive electronic search was performed in PubMed/MEDLINE, the Cochrane Library, and Google Scholar databases for appropriate published studies. The last search was conducted in January 2020. Two review authors assessed all studies identified by the search strategy and carried out data extraction.

**Results:**

Five studies were included in the systematic review which analysed a total of 280 patients with head and neck cancer. Methodological quality and outcomes were evaluated in every study included. The outcome measure was either subjectively assessed or objectively measured. Three studies used conventional TENS therapy to stimulate parotid glands which produced a significant increase in saliva production following therapy. Two studies used acupunctured TENS type to electrically stimulate acupuncture points scattered in the body and they reported improvement in saliva production at the same level as medical treatment. No reported adverse effect of TENS was identified.

**Conclusions:**

This systematic review confirms the safety and feasibility of TENS in the treatment of xerostomia. It is established that commencing daily TENS therapy simultaneously with radiotherapy has the most efficacy. Given the nonspecific parameters used in the included studies, further evidence is needed in order to establish optimal settings and parameters of TENS for treatment of xerostomia.

## Highlights

• Transcutaneous electrical nerve stimulation technique shows promise in treating xerostomia in patients with head and neck cancer.
• Concurrent radiotherapy treatment with TENS techniques in stimulating the salivary glands has a better outcome slowing the progress of developing severe dry mouth.
• Daily TENS technique treatment is required for optimal outcome in treating dry mouth.
• We recommend using a finite element mathematical modelling to design an optimal TENS parameters and effective electrodes geometries to enhance more selective stimulation of the nerve innervating the parotid glands.

## 1 Introduction

Head and Neck cancer (HNC) is diagnosed in over 12 000 people in the UK per year and more than 500 000 worldwide [1]. The overall incidence is increasing, with a change in the patient demographic, such that patients are younger at presentation with a greater chance of survival.

Radiotherapy remains one of the principal methods of treating HNC [2]. However, one of the long-term consequences of radiotherapy is a dry mouth, xerostomia [3]. It is generally accepted that the dose of radiotherapy used to treat HNC (55–70Gy) will damage saliva producing cells [4].

There are a number of factors that are important when considering the impact of radiation on saliva function. These include the total dose of radiation, whether radiation is unilateral, or bilateral, and the extent of the upper aerodigestive tract included in the fields [5]. Advanced techniques of tailored radiation, such as Intensity Modulated Radiotherapy (IMRT) or Volumetric Modulated Arc Radiotherapy (VMAT), may help spare key structures such as the major salivary glands [6]. The intrinsic patient response to radiotherapy is another important factor.

Dry mouth is reported as one of the most disabling long term symptoms experienced by survivors of head and neck cancer (HNC), and while IMRT has improved the ability to preserve salivary function by sparing exposure of the major salivary glands it remains a significant concern [5,7,8].

In a prospective study of post treatment symptoms of 107 HNC patients over a 12-month time period, dry mouth was rated as top priority at 3, 6 and 12 months after chemoradiation therapy. In a follow up study of 61 patients from the phase III PARSPORT trial using IMRT specifically to spare the salivary glands, dry mouth was still consistently the most important concern at all time points. Without IMRT, this figure more than doubles to 65% [9].

The aim of this systematic review was to provide an overview of evidence from previous studies on the effectiveness of electrical stimulations to increase saliva productions in radiotherapy and/or chemotherapy induced hyposalivation of head and neck cancer patients.

The primary outcome of interest is identifying the performance on saliva production by electrical stimulation in survivors of head and neck cancer patients.

The secondary outcome is a definition of a standardized clinical protocol and electrical stimulation parameters in order to improve saliva flow in survivors of head and neck cancer patients.

## 2 Methods

### 2.1 Protocol registration and eligibility criteria

This systematic review was carried out to evaluate the evidence for transcutaneous electrical stimulation of the salivary glands following hyposalivation induced by either radiotherapy or chemotherapy in head and neck cancer patients. The work has been reported in line with PRISMA (Preferred Reporting Items for Systematic Reviews and Meta-Analyses) [10] and AMSTAR (Assessing the methodological quality of systematic reviews) Guidelines [11]. This was to ensure high methodological rigour. This systematic review has been registered with the Research Registry and the identifying number is reviewregistry1027 [12].

#### 2.1.1 Studies were included when they met all of the following criteria

Included participants ≥18 years old with head and neck cancer who had undergone radiotherapy or chemotherapy; Included TENS treatment and had reported outcome in any type of saliva production methods.

Studies were published in English language.

#### 2.1.2 Exclusion criteria

Titles were unrelated to the keywords defined by the search strata.

### 2.2 Search strategy

A comprehensive search of MEDLINE (PubMed), EMBASE, and Google Scholar databases was performed on January 27, 2020. No restrictions on language nor publication date were applied. The MEDLINE search-string was as follows: (“ Head and neck cancer patient” OR ″ patients with head and neck caner) AND (“ hyposalivation” OR “Xerostomia” OR “Dry mouth”) AND (“radiotherapy induced” OR “radiation induced” or “Chemotherapy induced”) and (“Transcutaneous electrical stimulation” or “TENS” or “Electrical stimulation of Salivary glands”)

A guide stating the research question, search strategy, inclusion/exclusion criteria and risk of bias was formulated. The search and screening were performed by two of the authors (FS and SW), and disagreements were resolved by consensus.߭Data extraction was performed with the following information from each included study: Author, publication year, number, type of TENS used, duration of TENS used, and how salivary flow was measured.

## 3 Results

**Fig. 1:**
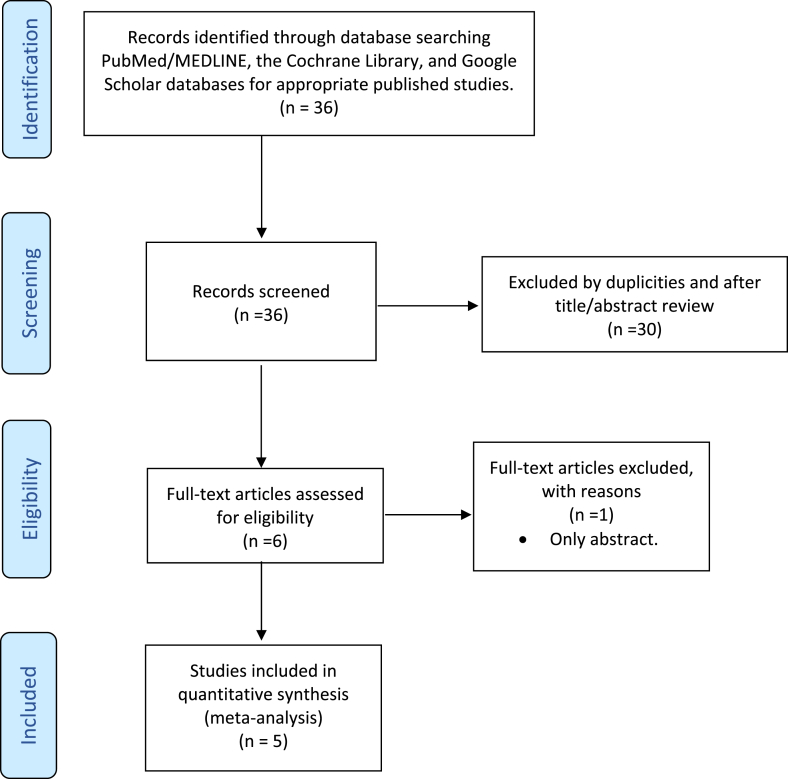
PRISMA 2009 flow diagram.

36 papers were identified using the search criteria, reducing to 28 after removal of duplications. 22 papers were excluded after reviewing the abstracts because they did not meet the eligibility criteria. 6 papers had a full text review. One of the papers was excluded because was only an abstract article and following this a total of 5 papers were included in this study. Fig. 1 shows a PRISMA diagram for this review.

Of the 5 papers identified three were designed as RCTs and two as interventional studies. In total across the 5 studies were included from Canada, USA, India, and Brazil. There were two approaches to stimulate salivary secretion identified. Two studies used the ALTENS (Acupuncture-Like Transcutaneous nerve Stimulation) approach, while 3 studies used Conventional TENS. The main results of the included papers are summarized in Table 1 and Table 2.

**Table 1:** Summary of included studies. (RCT = randomised control trial, Sp6, St36, CV24, LI4, St5 and P6 = acupuncture points).

Study and year	Study design	N	Stimulation parameters	Electrode position
Wong et al. 2015 [13]	RCT	96	• TENS - acupuncture (Codetron – 902-C) • Pulse width: 250 ms • Frequency: 4 Hz, Square pulses • Intensity: adjusted to produce a deep, strong, with or without mild aching, sensation at the attachment point of the electrodes. • 20 min per session	Sp6, St36, LI4 (active) CV24 (indifferent)
Vijayan et al. 2014 [14]	Interventional study	30	• TENS (MEDIHIGHTEC 8000 combo) • Pulse width: 250 μs • Frequency: 50 Hz, Square pulses • Intensity: defined as the maximum intensity that the subject still perceived to be comfortable. • 5 min per session	Parotid glands, bilaterally
Wong et al., 2003 [15]	RCT	37	• TENS - acupuncture (Codetron - 902-C) • Pulse width: 250 ms • Frequency: 4 Hz, Square pulses • Intensity: adjusted to produce a deep, strong, with or without mild aching, sensation at the attachment point of the electrodes. • 20 min per session	**Group A** Sp6, St36, LI4 (active) CV24 (indifferent) **Group B** Sp6, St36, P6 (active) CV24 (indifferent) **Group C** Sp6, St5 and 6, P6 (active) CV24 (indifferent)
Lakshman et al. 2015 [16]	Interventional study	40	• TENS (model-NS Electro Pulse) • Frequency: 500 Hz, sweep of 0.5–2 Hz. • AC pulses • Intensity: defined as the maximum intensity that the subject still perceived to be comfortable. • 10 min per session	Parotid glands, bilaterally
Paim et al. 2019 [17]	RCT	15	• TENS (Neurodyn II Ibramed®) • Frequency: 50 Hz • Pulse width: 250μs • Intensity: defined as the maximum intensity that the subject still perceived to be comfortable. • 20 min per session	Parotid and submandibular gland bilaterally

**Table 2:** Summary of output measures (VAS: Visual analog scale, XeQLOS: Xerostomia- Related Quality of Life Scale developed by University of Michigan.).

Study and year	Comparator	Subjective outcome measures	Objective outcome measures	Conclusion of the study
Wong et al. 2015 [13]	Pilocarpine	XeQLOS	Whole saliva produced • Spit method in a preweighed dry plastic container, conducted at baseline and at 4, 6, 9 and 15 months after the date of randomization	No significant difference in comparison to Pilocarpine. ALTENS was shown Superior due to lower toxicity
Vijayan et al. 2014 [14]	Before and after	Not assessed	Saliva flow • Spit method into a calibrated cup, collected during the test sessions.	TENS double the production of saliva
Wong et al. 2003 [15]	Pilocarpine	Five item xerostomia symptoms questioner with a VAS	Saliva flow • Spit method in a preweighed dry plastic container, performed at baseline and 6, 8, and 12 weeks after treatment began and at 3, 6, and 12 months after treatment completion.	Suggest that ALTENS treatment improve whole saliva production. Group A had the greatest improvement.
Lakshman et al. 2015 [16]	Healthy group	Not assessed	Salivary flow • Spit method collected in a glass beaker. Collected before and during the test sessions.	TENS increased salivary flow after daily use.
Paim et al. 2019 [17]	Control group	Self-perception of salivary flow (SPSF) and Quality of life (QL)	Salivary flow • Sialometry-using silicon sialagogue Collected before and immediately after each session.	The effect of the TENS lasted for 6 months after TENS therapy

ALTENS is a technique based on Chinese medicine, which use surface electrodes placed on acupuncture points instead of needle electrodes. This technique is characterized by using stronger and less habituating stimulation with lower frequency than conventional TENS. In particular these studies used a nonpolarizing, biphasic balanced square pulse of 250 ms duration delivered in trains with a repetition rate of 4 Hz. To deliver this type of stimulation they used a machine called Codetron, according to the authors [22,24] it provided comparable or better results than electroacupuncture treatment.

**Fig. 2:**
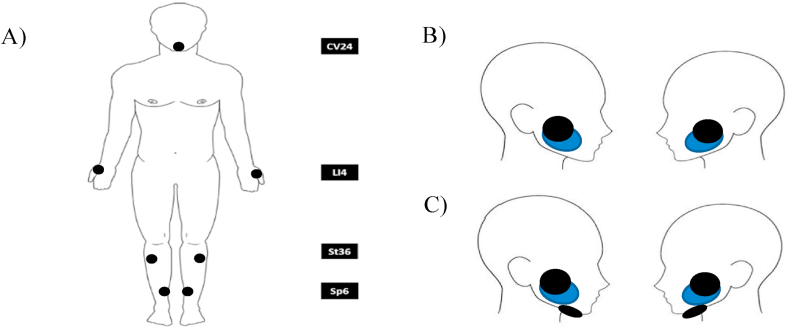
Electrodes Position used in the reviewed studies, A) ALTENS (22,36); B) Conventional TENS Parotid glands bilaterally and C) Conventional TENS Parotid glands plus Submandibular glands bilaterally.

Those studies using a Conventional TENS approach, they used the same parameters of 50 Hz frequency and a pulse width of 250μs square pulses and 1 study used an AC pulse of 500 Hz delivered with a repetition of 0.5–2 Hz. All the studies reported using commercial TENS machines. The criteria to define the current intensity of stimulation in all the studies was according the comfort of the subjects, just Paim et al. [26] reported the current intensity tolerated by patient during intervention which was 38.8 ± 7.5 mA. All the studies that used TENS approach placed the electrodes over the salivary glands, two studies placed the electrodes over the parotid gland bilaterally (Fig. 2B) and one study also placed an electrode over the submandibular gland (Fig. 2C). For ALTENS approach the best position of the electrodes was Sp6, St36, LI4 points as active electrodes and CV24 point as indifferent electrode (shown in Fig. 2A), this was determined in Ref. [24] where they found that Group A had the greatest improvement in saliva production.

In terms of protocols, we found differences among studies. Wong et al. [22,24], in their studies defined 24 ALTENS sessions, 20 min each, two sessions per week over 12 weeks. Vijayan et al. [23] and Lakshman et al. [25], used simpler protocols, with sessions of 5 min and 10 min respectively. Paim et al. [26], instead, used 8 sessions of 20 min each, twice a week over 4 weeks. All studies asked to the patients to refrain from chewing gum, smoking, and to avoid oral hygiene procedures for at least 1 h before the appointment. There was a wide variety regarding the duration of stimulation and length of the treatment. The most intense protocol involved daily use for up to 6 months after radiotherapy. None of studies reported on long term outcome of the treatment.

All the studies used different outcome measures, which are shown in Table 2. Three of studies used both subjective patients and objective reported outcome measures. While two used only objective measures of salivary output.

Four of the five studies used a saliva flow spit method to measure output. The spit method measures the accumulation of saliva in the floor of the mouth and during a defined period, spit into a pre-weighed or graduated test tube. One study [26] proposed a sialometry technique using a Halitus® kit (Toiletries Ltda.) to measure saliva output. Here participants were instructed to chew a silicone sialogogue for 5 min and place all saliva produced into a collection tube. To precipitate the foam and convert it into saliva, dimeticona was used. Each drop of dimeticona corresponded to 0.02 mL, and this amount was subtracted from the final volume. In all the studies, the saliva flow was calculated on a per minute rate.

Overall, the studies all reported an increased saliva production. Vijayan et al. [21] group reported that the saliva production was approximately doubled after TENS therapy. They reported an increase in saliva production from a mean unstimulated rate was 0.056 mL/min and the mean of stimulated group was 0,12 mL/min. Wong et al. [22] compared acupuncture like TENS with Pilocarpine, and they found both improved saliva production, however, Pilocarpine was found inferior to TENS due to its significant toxicity. Paim et al. [26] reported that conventional TENS increased the self-perception of saliva flow rate by 96%, which was verified with sialometry, resulting in a median gain of 260% after 8 weeks. Lakshman et all [25] reported that using TENS daily during the full course of radiotherapy increment the saliva flow between unstimulated and stimulated in 93% at zero week (0.84 mL/min to 1.62 mL/min), and in 146% at third week (0.56 mL/min to 1.38 mL/min). No studies reported any adverse outcomes from salivary stimulation.

## 4 Discussion

Dry mouth is caused by a wide variety of causes, including autoimmune conditions such as Sjorgens, diabetes and radiotherapy for head and neck cancer. For those patients with a dry mouth following radiotherapy a number of pharmacological and non-pharmacological treatments have been suggested.

Saliva is produced from the major and minor salivary glands. It is estimated that up to 90% is produced from the major salivary glands. Unstimulated saliva production is predominantly from the submandibular glands which are responsible for approximately 60%. The remaining 20–25% from the parotids and 7–8% form the lingual and the remainder from the minor salivary glands. This is in contrast to the stimulated saliva production which is from the parotid (60%), SMG and the remainder from the lingual/minor salivary glands [[18], [19], [20]]. Control is mediated via the autonomic nervous system. Saliva is produced at a rate of 0.3-0.7 mL/min when unstimulated and increases to 1.5–2 mL/min when stimulated. Hyposalivation is defined as a resting salivary flow of less than 0.1 mL per minute or less than 0.5–0.7 mL per minute when salivary glands are stimulated.

Several reviews have considered the benefit of pharmacological agents and to date there is no high quality evidence that topical agents can improve dry mouth [21]. The most recent Cochrane review in 2017 looking at pharmacological interventions for preventing dry mouth following radiotherapy concluded that there was some low‐quality evidence to suggest that amifostine prevents the feeling of dry mouth in the short term [22]. However, they concluded that it is less clear whether or not this effect is sustained to 12 months postradiotherapy. They also concluded that the benefits of amifostine should be weighed against its high cost and side effects and that there was insufficient evidence to show that any other intervention was beneficial. Mercandante et al. concluded that pilocarpine and cevimeline should be first line of therapy for patients with in radiotherapy-induced xerostomia and hyposalivation [23]. TENS is superior to pilocarpine because its effect lasts much longer, it has no chemical toxicity and fewer contraindications to its use [13,17].

Non-pharmacological devises such as TENS have been suggested as a possible solution for several years. TENS is a non-invasive technique which conveys pulsed electrical currents across the intact surface of the skin to activate underlying nerves. Following the publication of Melzack and Wall's “Pain Mechanisms: A New Theory” TENS became increasingly popular as a non-pharmacological option for treating pain [24]. Different TENS techniques are used to selectively activate populations of nerve fibres to elicit mechanisms leading to pain relief. They are commonly divided into three different categories (Table 3) based on the charge delivered with each pulse (intensity) of current and the frequency of the pulses used to relieve pain [[25], [26], [27]].

**Table 3:** Summary of TENS devices classifications.

Conventional TENS	Activation of large diameter non-noxious afferents Surface electrodes	Low-intensity stimulation/high-frequency (between 10 and 200 pulses per second)
Acupuncture-like TENS	Activate small diameter motor fibres, producing muscle twitches. Needle or surface electrodes.	High-intensity stimulation/low-frequency (less than 10pps, usually 2–4 pulse per second)
Intense TENS	Activate small diameter noxious afferents. Eliciting peripheral nerve blockade and extra-segmental analgesia Surface electrodes	High-intensity stimulation/high-frequency (up to 200 pulse per second)

A Cochrane review published in 2013 concluded that there is insufficient evidence to determine the effects of electrostimulation devices on dry mouth symptoms [28]. However, the study data included only a small proportion of radiation induced dry mouth.

TENS has been suggested as a way of treating dry mouth caused by a number of other aetiologies, including diabetes and Sjorgens syndrome [29,30].

Dry mouth following radiotherapy for the treatment of head and neck cancer remains a significant problem for patients, this is despite changes to radiotherapy techniques deigned to minimise radiotherapy to the major salivary glands [3,31]. The SALRISE (Salivary electro-stimulation for the treatment of dry mouth in patients with Sjogren's syndrome: a multicentre randomised sham-controlled double-blind study) is looking at TENS stimulation in Sjogren's syndrome [32].

Most of the studies presented in this review suggest that there a benefit in producing saliva by stimulating the salivary glands. However there a number of parameters that have yet to be defined including which glands to stimulate, the type of TENS technique to be applied, the frequency and intensity of the stimulation current, the type, size and placement of the stimulating electrodes, and the frequency and duration of stimulation sessions to provide clinical improvement. Regarding to placement of the electrode, external placement may be impacted by radiation damaged skin, while intra-oral placement in patients treated with radiotherapy is problematic due to the local side effects of the radiotherapy. Furthermore, all of the studies used different end points to assess results. Both subjective and objective measures were used.

Electrical stimulation of the parotid glands can be performed locally via the conventional method or via acupuncture/distance stimulation. The latter method with inhibition of the sympathetic and stimulation of parasympathetic was found to have a less impact on saliva production in comparison to conventional method [13,15]. Conventional TENS method is designed to stimulate a bigger segment of the skin whereas acupuncture like method is fundamentally designed to stimulate the nerve instead of the gland itself. Another unclear parameter which remains untested in these studies, is frequency and duration of conventional TENS usage. All three conventional studies used different durations of TENS application. None of the studies reported on adverse events such as pain, discomfort, or skin reactions to the surface electrodes.

### 4.1 Limitations and challenges of this study

The limitations of this systematic review are the literature search was limited to articles published in English. No other pathologies involve on the decrease of saliva production was considered. Further prospective study with larger number of cases should be perform. The differences and inconsistencies on the duration and type of electrical stimulation used by researchers, it made difficult to reach any conclusion with respect to stablish what is the best strategy to use the electrical stimulation for saliva production. The positive findings of all studies mean publication bias must be considered as a potential limitation.

## 5 Conclusion

TENS has been shown to increase the salivary flow in patients with head and neck malignancy following radiotherapy and/or chemotherapy. However, as we mentioned at the discussion, it is not clear which type of TENS technique and what parameters are the optimal and most beneficial for this application. This opens a good opportunity for researchers to carry on long-term studies and define a clinical and technical standard of electrical stimulation for saliva production. We recommend using mathematical modelling such as Finite Element Models (FEM) for designing future studies involves TENS technique. This type of modelling has been explored for the last decades to define the most optimal electrical stimulation parameters such as, current intensity levels, pulse waveform and type of stimulation in many other applications of TENS machines [29](30).

3D MRI of the head and neck models in combine with axon models such as MRG axon model [28], which represent the auriculotemporal nerve, it provides better understanding of what type of electrical parameters are necessary to activate the nerve and hence, increasing the saliva production. In all these reviewed studies, they used the conventional commercial TENS electrodes to deliver the stimulation, however, we believe that is necessary to evaluate electrode designs in terms of geometries and material.

## Ethical approval

N/A.

## Sources of funding

This work was supported by grant 10.13039/501100002850FONDECYT 3180551 from the National Agency of research and development (ANID) - Chilean Government.

## Author contribution

Dr Fatemeh Salimi (First Author) , data design, data collection, data analysis or interpretation and writing the paper.

Dr Francisco Saavedra (Joint First Author) data design, data collection, data analysis or interpretation, writing the paper.

Prof Brain Andrews, concept of the study.

Prof James FitzGerald, concept of the study.

Prof Stuart C Winter concept of the study.

## Registration of research studies

1. Name of the registry: ResearchRegistry.com
2. Unique Identifying number or registration ID: reviewregistry1027
3. Hyperlink to your specific registration (must be publicly accessible and will be checked):
4. https://www.researchregistry.com/browse-the-registry#registryofsystematicreviewsmeta-analyses/

## Guarantor

Dr Fatemeh Salimi.

Dr Francsico Saavedra.

## Consent

N/A.

## Provenance and peer review

Not commissioned, externally peer reviewed.

## Declaration of competing interest

No conflict of interest.
